# Prioritizing removals of a highly cryptic invasive species using machine learning and demographic weighting

**DOI:** 10.1002/eap.70317

**Published:** 2026-09-21

**Authors:** Alexander S. Romer, Melissa A. Miller, Sergio A. Balaguera‐Reina, Michelle C. Bassis, Brandon Welty, Michael Kirkland, Amy Peters, Edward F. Metzger, Jenna Cole, LeRoy Rodgers, Frank J. Mazzotti

**Affiliations:** ^1^ Department of Wildlife Ecology and Conservation, Fort Lauderdale Research and Education Center University of Florida Davie Florida USA; ^2^ South Florida Water Management District West Palm Beach Florida USA

**Keywords:** artificial intelligence, boosted regression trees, Burmese python, contractor performance, ecological modeling, everglades ecosystem, generalized additive models, invasion ecology, predictive analytics, reproductive weighting, reptile demography, survivorship curve

## Abstract

Invasive species present substantial challenges to biodiversity conservation, particularly when low detectability and demographic heterogeneity obscure the population‐level consequences of management actions. Invasive Burmese pythons (*Python bivittatus*) in South Florida exemplify this challenge, as metrics based on raw removals or body size alone may not reflect demographic impacts. To address this, we introduce the Weighted Removal Index (WRI), a demographic weighting framework that prioritizes removals based on expected contributions to survival and reproduction. Using survey data from the South Florida Water Management District's Python Elimination Program, we quantified operational and environmental conditions associated with higher survey WRI, defined as the summed WRI of all pythons captured on a single survey, and evaluated three forecasting approaches: Generalized Additive Models (GAMs), Boosted Regression Trees (BRT), and Neural Networks (NN). Survey WRI was highest under waning lunar phases, when surveys were assisted, and under warmer and more humid conditions. Additionally, survey WRI was elevated on warm days early in the wet season and cool dry‐season days. In held‐out test evaluation, the NN achieved the highest balanced accuracy for predicting survey success (0.703), and the lowest error for predicting survey WRI (root mean squared error [RMSE] = 5.50). Contractor performance was highly heterogeneous, with contractor‐specific effects positively associated with mean survey WRI (*R* ^2^ = 0.589) and capture rate (*R* ^2^ = 0.596). Notably, model‐based predictions of survey success fell within the upper tail of the contractor capture‐rate distribution, indicating that near‐term forecasting can match the performance of the highest performing contractors. Together, demographic weighting and near‐term forecasting provide a practical decision‐support framework for guiding effort toward high‐impact removals in cryptic invader management.

## INTRODUCTION

The proliferation of invasive species presents a significant challenge to preserving biodiversity and ecosystem function (Linders et al., 2019; Pejchar & Mooney, 2009). Invasive species threaten 14% of critically endangered terrestrial vertebrates (Dueñas et al., 2021). Furthermore, 20% of animal extinctions are directly attributed to invasive species, and they are partially implicated in an additional 34% (Clavero & García‐Berthou, 2005). Invasive species also incur substantial economic costs by reducing ecosystem services (Walsh et al., 2016), damaging infrastructure (Connelly et al., 2007), and disrupting agriculture (Paini et al., 2016). Between 1960 and 2020 in the United States, the indirect and direct costs of invasive species totaled $4.52 and $1.22 trillion, respectively (Fantle‐Lepczyk et al., 2022). Thus, control of invasive species is typically an important objective for organizations involved in biodiversity conservation and natural resource management.

Biological invasions are characterized by high rates of proliferation, rapid geographic spread, and a competitive advantage over native species (Valéry et al., 2008). Consequently, populations of invasive species increase in density and spatial extent following their initial introduction, eventually leading to the establishment of a self‐sustaining population (Sakai et al., 2001). Effective management of invasive species thus requires a dynamic approach. Surveillance should be employed to detect the presence and extent of invasion, with control actions continuously adjusted accordingly (Epanchin‐Niell, 2017). In the early stages of invasion, removal techniques that are effective at low population densities are preferred, as they increase eradication potential and reduce long‐term costs (Epanchin‐Niell, 2017; Kaiser & Burnett, 2010). However, once an invasive species becomes established and eradication is no longer feasible, long‐term management typically shifts to control and mitigation (Epanchin‐Niell, 2017). Strategies to achieve these objectives include implementing cost‐effective control techniques, encouraging stakeholder participation, and prioritizing the spatial and temporal scope of control efforts to maximize impact (Epanchin‐Niell, 2017). Additionally, the ecological and geographic context of invasion should be considered when designing management actions, as in the case of South Florida.

South Florida is bordered by ocean to its south, east, and west and is geographically isolated from other subtropical climates by surrounding temperate regions, which experience cooler seasonal temperatures (Lodge, 2016). This geographic isolation has led to unique biotic assemblages in South Florida, which have historically been isolated from taxa endemic to other subtropical ecosystems. Isolation is associated with greater invasibility, or vulnerability to biological invasion (Denslow, 2003; Lonsdale, 1999; Moser et al., 2018). This pattern has resulted in disproportionate effects of invasive species on insular communities (Doherty et al., 2016). Similarly, South Florida's ecosystems display a high degree of invasibility. The State has the highest richness of introduced reptiles and amphibians in the United States (Krysko et al., 2011). In addition to its geographic setting, the prevalence of invasive herpetofauna in South Florida is attributed to the pet trade (Krysko et al., 2011) and extreme weather events that facilitate escape (Engeman et al., 2011), human introduction for biological pest control (Wilson & Johnson, 2017), and accidental introductions as cargo stowaways (Hardin, 2007). Given the diversity and abundance of invasive reptiles in Florida, prioritization of management efforts is required.

Among Florida's invasive reptiles, Burmese pythons (*Python bivittatus*) are notable for their significant impact on native wildlife. Burmese pythons exhibit an exceptionally broad dietary niche, consuming both native and introduced vertebrates (Guzy et al., 2023). Prior to 2000, mammals were consistently observed in Everglades National Park during nighttime road surveys (Dorcas et al., 2012). However, from 2003 to 2011, observations of several mammal species declined by 87.5%–100%, coinciding temporally and spatially with the proliferation of pythons (Dorcas et al., 2012). Furthermore, Taillie et al. (2021) demonstrated that medium and large‐bodied mammals, such as marsh rabbits (*Sylvilagus palustris*) and white‐tailed deer (*Odocoileus virginianus*), experienced declining occurrences along the invasion front. Marsh rabbits, once a keystone species in the Everglades, have been extirpated in many locales due to predation from pythons (McCleery et al., 2015; Sovie et al., 2016). Wading birds (Pelecaniformes) constitute a substantial portion of python's diet in Florida, making up 20.8% of identified prey items (Guzy et al., 2023). Of particular concern is predation of two state threatened (little blue heron [*Egretta caerulea*], roseate spoonbill [*Platalea ajaja*]) and a federally threatened species (wood stork [*Mycteria americana*]) (Guzy et al., 2023).

In addition to their direct impacts, pythons generate substantial indirect ecological effects. Infections in native snake species by a non‐native lung parasite, the pentastome *Raillietiella orientalis*, are attributed to spillover resulting from pythons (Miller et al., 2018, 2020). Vertebrate communities impacted by python predation exhibit altered patterns of urban habitat use (Reichert et al., 2017), increased potential for zoonotic disease transmission (Burkett‐Cadena et al., 2021; Hoyer et al., 2017), and compensatory shifts in dietary niches (Willson, 2017). Due to their substantial effects on native ecosystems, Burmese pythons are subject to intensive management.

The ecology and life history of Burmese pythons present challenges to efficient removal. Burmese pythons are large‐bodied (up to 5–6 m total length [TL]) ambush predators (Currylow et al., 2022a; Easterling & Bartoszek, 2019; Guzy et al., 2023). Due to their hunting strategy, they spend a high proportion (86%) of their time at rest (Whitney et al., 2021), often submerged (Smith et al., 2021). These behaviors reduce detectability, which is estimated between 1 and 5% (Dorcas & Willson, 2013; Nafus et al., 2020). Furthermore, pythons exhibit a type III survivorship curve with high mortality rates early in life and low mortality rates at maturity. Juvenile pythons have a two‐year survivorship of 7.1% (Pittman & Bartoszek, 2021) while annual adult survival has been modeled at 90% based on expert opinion (Willson et al., 2011). Thus, reproductive adults are likely to persist on the landscape without human intervention. Mature female pythons produce an average clutch size of 34 eggs, with a maximum recorded size of 96 (Currylow et al., 2023). As with other large‐bodied reptiles, juvenile pythons are expected to comprise a larger proportion of the population than mature individuals (Briggs‐Gonzalez et al., 2017). Demographic modeling of sea turtle populations, which share similar life history characteristics, has demonstrated that some life stages contribute disproportionately to population vital rates (Crouse et al., 1987; Mazaris et al., 2005). Thus, the impact of removing a python on population vital rates likely depends on its demographic characteristics. To enhance the impact of python removal efforts, two key challenges must be addressed: (1) increasing the ability of natural resource managers to locate pythons and (2) prioritizing removals to maximize population impacts.

To facilitate the management of Burmese pythons, South Florida Water Management District (SFWMD) and the Florida Fish and Wildlife Conservation Commission (FWC) have implemented contracted removal programs. In 2017, both FWC [Python Action Team Removing Invasive Constrictors (PATRIC)] and SFWMD [Python Elimination Program (PEP)] initiated removal programs in which skilled contractors are employed to remove pythons from state and federal properties. As of February 2024, contractors have removed 13,136 out of the 21,006 total pythons removed (63%) from Florida (Chejanovski, 2024; Florida Fish and Wildlife Conservation Commission, 2024). Additionally, these state‐managed python contractor programs collect data on survey effort, conditions, outcomes, and python demographics, which can be used to address contemporary challenges with python management.

Models of invasive species control suggest demographic removal can accelerate population suppression compared to strategies focused solely on the invasion core or front (Harris et al., 2009). Additionally, population suppression of Brown treesnakes (*Boiga irregularis*) demonstrated that removal of large reproductive females has the largest impact on population vital rates (Nafus et al., 2022). Therefore, developing a demographic weighting scheme for pythons would allow removals of key demographics to be prioritized, increasing efficacy of management efforts and reducing resources. Furthermore, the low detectability of pythons decreases the efficiency of contractor surveys (0.09 pythons/h; McCaffrey et al., 2022). However, the application of machine learning approaches, including Boosted Regression Trees (BRTs), Generalized Additive Models (GAMs), and Neural Networks (NNs), is enhancing predictive capacity in ecological applications. BRTs have been applied to model the distribution and abundance of ticks, outperforming traditional linear models and leading to improved predictions for public health interventions (Manley et al., 2023). GAMs incorporating spatial autocorrelation and delayed environmental effects improved tuna distribution forecasting by 43%, enhancing fisheries management predictions (Salazar et al., 2021). NNs recognize complex patterns and relationships within large datasets, making them increasingly valuable for ecological applications (Brodrick et al., 2019). Consequently, NNs have been utilized to detect amphibian species in water reservoirs, supporting ecological city planning (Karapinar Senturk, 2022). NN analysis of frog calls has improved monitoring of reproductive phenology and species identification (Kimura & Sota, 2023). Consequently, machine learning approaches should be investigated as a method to increase python detectability and, thereby, removals.

In this study, we developed and evaluated a Weighted Removal Index (WRI) which approximates the effect of individual python removals on population vital rates. This is accomplished by scaling the size of captured individuals according to their survivorship and reproductive output. Our research aim is to aid managers in prioritizing removal of individuals that likely contribute disproportionately to population growth and persistence. Specifically, our objectives were to: (1) develop the WRI to inform management of invasive pythons, (2) compare the performance of various model frameworks, including GAMs, BRT, and NNs, in forecasting survey success and WRI, and (3) analyze contractor performance to quantify variability in survey outcomes and establish a baseline for evaluating machine learning approaches. To accomplish these objectives, we leveraged demographic removal and survey outcome data collected by a state‐funded contractor removal program (SFWMD PEP).

We predict that WRI will be influenced by environmental conditions (e.g., air temperature) and temporal variables (e.g., Julian day of year) that correspond with the life history of pythons (McCaffrey et al., 2025). For example, we expect that total WRI of surveys will increase during the breeding season when aggregations of adult pythons may be encountered by contractors. We also predict that NNs will outperform GAMs and BRTs in predictive capacity for survey success and survey WRI (sum of WRI for all pythons captured on a single survey). While GAMs and BRTs are widely used for ecological modeling, NNs may capture higher order feature interactions and dependencies in python capture demographics and survey covariates, which may be less effectively parameterized by GAMs and BRTs. Finally, we expect that contractor identity will significantly affect survey WRI due to variations in skill, experience, and search techniques.

## METHODS

### Study area and data collection

Data on Burmese python survey effort and removals were obtained from SFWMD PEP. Surveys for the PEP are conducted within designated management properties in the Greater Everglades Ecosystem, an expansive wetland characterized by a subtropical climate and unique habitats differentiated by microtopography and hydrology (Lodge, 2016). This dataset includes detailed records of survey effort, outcomes, and demographic information on removed pythons, such as sex and snout‐vent length (SVL).

The original dataset was screened and reformatted to ensure accuracy and independence of survey records, following the procedures detailed in McCaffrey et al. (2025). Data collection was conducted by contractors who manually input survey metadata into an ArcGIS Survey123 form (ESRI, 2023), while their locations were automatically recorded using the ArcGIS Field Maps application (ESRI, 2024). Variables such as contractor identity, survey start and end times, python captures, the presence of assistants, and whether the survey was a rapid response to python sightings were recorded. GPS tracking data was used to validate contractor‐submitted survey records. Survey records were retained for analysis only if the start and end times from Survey123 matched those generated by the GPS data or if discrepancies could be accurately corrected. Tracking sessions with poor GPS accuracy (median horizontal accuracy >100 m) or those occurring outside approved survey properties were excluded. Independent survey events were defined using criteria based on time and distance between consecutive tracking points. Surveys were removed if they were missing more than 15 min of tracking data or if there was a discrepancy of 30 min or more between the start or end time of a survey entry. The final dataset consisted of 4092 independent survey events which were conducted between 27 May 2020 and 30 April 2022, totaling 16,336.75 survey hours and 1556 python removals.

Missing SVL values (*n* = 656) were imputed using predictions from a GAM with sex and TL as predictors. The model was fit to a random 75% subset of captures with observed SVL values (*n* = 657), while the remaining 25% (*n* = 217) were reserved for model evaluation. The model employed a gaussian distribution with a log link. Model performance is reported in the results.

Environmental data for the survey period were collected from SFWMD's DBHydro meteorological monitoring stations located near approved survey lands (South Florida Water Management District, 2025). For each survey, the most proximate weather station was used to obtain environmental variables, with priority given to stations capable of recording all variables of interest. If the nearest station could not provide complete data, the next closest station which could provide complete data was used. Lunar illumination data for each survey were obtained using the R package “lunar” (Lazaridis, 2022). Daily 1991–2020 air‐temperature climate normals for Miami International Airport, Florida, were obtained from NOAA's U.S. Daily Climate Normals dataset through the National Weather Service NOWData interface (Palecki et al., 2021). All statistical analyses were performed using R version 4.3.3 (R Core Team, 2024).

### Development of the weighted removal index

We developed the WRI to quantify the relative impact of removing individual Burmese pythons based on easily measured demographic characteristics. The WRI integrates several biological factors that influence population vital rates, namely, survival and reproduction. SVL in Burmese pythons is associated with important aspects of their reproductive biology, including maturation and egg production (Currylow et al., 2022a; Willson et al., 2014). Additionally, survivorship is believed to be highly variable according to size class. Pittman and Bartoszek (2021) found only 7.1% of juveniles survived 3 years post‐hatching, while Willson et al. (2011) modeled an annual adult survivorship of 90% based on expert opinion. Therefore, we modeled survivorship as a sigmoidal function of SVL (measured in cm) corresponding to a type III survivorship curve. This survivorship function is given by

$$\text{Surv}\left(\mathrm{SVL}\right)=\frac{0.95}{1+e^{-\;0.05\times \left(\mathrm{SVL}-150\right)}}$$

This function models survivorship such that juvenile pythons experience initially high mortality rates, which decline exponentially with growth, eventually approaching an asymptotic maximum survivorship (0.95) at adult size. The parameter 150 cm corresponds to the inflection point at which survivorship of pythons is modeled as 0.5. The rate parameter of the sigmoidal function, 0.05, controls the steepness of the curve around the inflection point, determining how quickly survivorship transitions from low juvenile survivorship with growth. Raw survivorship values are converted to survivorship weights (*w*_surv_) by multiplying with an arbitrary scaling factor of 10:

$$w_{\text{surv}}=10\times \text{Surv}\left(\mathrm{SVL}\right)$$

This is performed to balance the relative contributions of survivorship and reproduction in the final WRI calculation.

In addition to survivorship, population vital rates are sensitive to variation in potential reproductive output between individuals. Consequently, when individuals reach sexual maturity (determined using SVL cutoffs), a reproductive weight is calculated to model this effect. We use distinct reproductive weighting schemes for male and female individuals to account for sexual dimorphism and imbalanced reproductive investment associated with large constrictors (Reed & Rodda, 2009). Female pythons typically reach sexual maturity at an SVL of ~260 cm, at which point they become capable of reproducing (Reed & Rodda, 2009; Willson et al., 2011), although smaller reproductive females have been noted (Currylow et al., 2022a, Willson et al., 2014). Therefore, reproductive weights are only calculated for female pythons with an SVL ≥260 cm. Additionally, female reproductive output is closely tied to body size, with larger females capable of producing more eggs. This potential egg production was estimated using empirical data from Currylow et al. (2022a, 2022b), which reported size‐specific fecundity rates. We slightly modified the equation from Currylow et al. (2022a) for predicting oviductal eggs of mature females by transforming SVL and oviductal egg production into the logarithmic scale before fitting the regression model to improve linearity of the relationship. The reproductive weight for females (*w*_rep♀_) is calculated using the formula:

$$w_{\mathrm{rep}♀}=\left\{\begin{array}{cc} \frac{e^{2.168\times \log\left(\mathrm{SVL}\right)-8.920}}{2} & \text{if}\;\mathrm{SVL}\geq 260\;\&\;S=♀\\ 0 & \text{if}\;\mathrm{SVL}<260\;\&\;S=♀ \end{array}\right.$$

Thus, we model reproductive weights for females using the conceptual framework that female pythons reach sexual maturity at an SVL of approximately 260 cm, and reproductive output increases exponentially with size. An arbitrary scaling factor of ½ is used to balance this term with *w*_surv_ and *w*_rep♂_.

Male pythons typically reach sexual maturity at an SVL of ~200 cm (Currylow et al., 2022a, 2022b) which we use as our SVL cutoff for assigning male reproductive weights. Male body size is positively associated with reproductive success in several species of snakes. In European grass snakes (*Natrix natrix*), larger males were involved in more successful mating events than smaller males (Luiselli, 1996; Madsen & Shine, 1993). In red‐sided garter snakes (*Thamnophis sirtalis parietalis*), larger males secured more mating events and paired with larger, more fecund females, highlighting the importance of male body size even in species lacking overt male–male combat (Shine et al., 2000). Consequently, we assign reproductive weighting to male pythons using population‐level statistics as there are no studies relating male size and reproductive success in this species. We use the SD of SVL within the population of mature males, using the formula:

$$w_{\mathrm{rep}♂}=\left\{\;\begin{array}{cc} \max\left(0,\frac{\mathrm{svl}-231.6}{29.3}\right) & \text{if}\;\mathrm{svl}\geq 200\;\&\;S=♂\\ 0 & \text{if}\;\mathrm{svl}<200\;\&\;S=♂ \end{array}\right.$$

This calculation represents the idea that large mature males will be more successful in competing for mates. There are two important caveats to *w*_rep♂_. First, males do not receive a reproductive weight until they reach the average SVL of mature males (μ = 231.6, σ = 29.3). This is unlike females who receive a reproductive weight immediately upon reaching maturity. As described above, male mating success is likely a function of body size rather than simply biological capacity; hence this implementation. Second, *w*_rep♂_ scales linearly with SVL, which is the same relationship between male body size and mating success recovered by Madsen and Shine (1993).

WRI is calculated by summing the survivorship weight and the reproductive weight, depending on the sex of the python. The formula for WRI is as follows:

$$\mathrm{WRI}=w_{\text{surv}}+\left\{\begin{array}{cc} w_{\mathrm{rep}♀} & \text{if}\;S=♀\\ w_{\mathrm{rep}♂} & \text{if}\;S=♂ \end{array}\right.$$

By integrating survivorship and reproductive information, the WRI provides a method of weighting individuals in a manner that approximates their contribution to population dynamics.

### Statistical analysis

We applied various modeling approaches to predict survey outcomes, including both the probability of capturing at least one python and the total WRI of removals from a survey. We also assessed the impact of environmental and operational variables using this metric, thereby evaluating its utility for management.

We used survey metadata (Appendix S1: Table S1) to predict the summed WRI values for all pythons removed on a survey, hereafter referred to as “survey WRI.” This was performed using three modeling approaches: GAMs, BRT, and a dual‐model NN pipeline. GAMs offer high interpretability, making them useful for understanding how specific variables influence WRI, but they may be limited in capturing complex relationships (Welchowski et al., 2021; Zhang et al., 2024). BRTs improve predictive performance by capturing nonlinearities and interactions, though at the cost of reduced interpretability (Austin, 2007). NNs, which deliver exceptional predictive capacity, operate as “black boxes,” where the relationships between inputs and outputs are difficult to explain in simple terms (Pichler & Hartig, 2023; Thessen, 2016). The combination of these models was chosen to leverage the interpretability of GAMs, the flexibility of BRTs, and the predictive power of NNs, creating a comprehensive framework for evaluating the factors influencing and predicting survey WRI. We utilized the R packages “mgcv,” “gbm3,” and “keras” for GAM, BRT, and NN modeling, respectively (Allaire & Chollet, 2024; Hickey et al., 2024; Wood, 2017).

For the NN model, predicted survey success served as an input to the WRI model as part of a hurdle‐style architecture described below (Kong et al., 2020). Comparable formulations were not implemented for GAMs or BRTs because stacked pipelines are not conventional for these models and would reduce interpretability. For example, introducing an additional first‐stage model would complicate standard inference approaches, such as variable importance or partial effect estimation (Elith et al., 2008; Pedersen et al., 2019).

Model development and evaluation were conducted using a system of data partitions: training (75% of samples), validation (12.5%), and test (12.5%). The distribution of WRI was maintained in each data split using the *createDataPartition* function from the “caret” package (Kuhn, 2008). Maintaining WRI distribution across splits was crucial to ensure that the model encountered a representative range of WRI values during training, validation, and testing. The training set was used in all model fitting and training procedures. The validation set was used to fine‐tune model hyperparameters, such as through hyperparameter tuning using Bayesian optimization. The test set was withheld from all model fitting procedures and was used exclusively to evaluate each model's performance.

To evaluate model performance in predicting survey success, we fit each model class using a binary response variable which indicated whether at least one python was captured on a survey. Models of survey success generated predicted values on the probability scale. Thus, for each model, we applied a classification threshold of 0.5 when calculating performance metrics. Cross‐model comparisons of survey success and WRI predictions were conducted using the test dataset to assess the predictive capacity of the models and their ability to generalize to new data. Survey success models were not used for inference as the impact of environmental and operational conditions on unweighted python removal was thoroughly examined in McCaffrey et al. (2025).

### Generalized additive models

We fit two GAMs to evaluate survey outcomes as measured by survey success and survey WRI. Both models utilized the same formula specification (Appendix S1: Equation S1) which contained a suite of environmental, temporal, and operational variables. Continuous environmental predictors, such as air temperature, wind speed, and humidity, were modeled using thin‐plate splines, which include a smoothness penalty term. We employed automatic smoothness selection to reduce the risk of overfitting. As such, in cases where the data did not support a complex nonlinear relationship, the model reduces smooth terms to a linear function or to no effect. Cyclical phenomena, such as Julian day and start time, were modeled using cyclic cubic splines to account for their periodicity. Interactions between variables, such as Julian day and air temperature, were modeled using tensor product splines. Operational predictors included survey duration, transport method used during the survey, whether the contractor was assisted, and survey interval (the interaction between survey duration and start time). Random effects for categorical variables, such as contractor identity and survey properties, were used to account for unobserved heterogeneity. All random‐effect smooths were estimated jointly within the GAM framework, so coefficients reflect the marginal contribution of each factor after accounting for shared variance with other effects (Wood, 2017).

For the survey success GAM, we modeled the probability of capturing at least one python using a binomial distribution with a logit link. For the survey WRI GAM, we evaluated multiple candidate families, including Gaussian, Gamma, negative binomial, Tweedie, and Scaled‐t distributions. Each family was paired with relevant link functions (e.g., identity, log, inverse), and model performance was assessed using deviance explained and residual plots generated by the *appraise* function from the package “gratia” (Simpson, 2024). All models were fit with the training dataset and the *bam* function from the package “mgcv” (Wood, 2017).

### Boosted regression trees

BRTs were used to model both survey success and survey WRI. BRTs iteratively combine many shallow decision trees, with each successive tree fit to the residuals of the previous, thereby enhancing predictive accuracy (De'ath, 2007). Survey success was modeled using the Bernoulli distribution, while the Tweedie distribution was used to accommodate the zero‐inflated nature of survey WRI.

Both BRT models used the same set of environmental, temporal, and operational predictors described above. Hyperparameters governing model complexity and fitting were tuned using a grid search applied to the training data. This process explored variations of the number of trees, interaction depth, learning rate, minimum observations per terminal node, and a weighting factor for capture events. Model performance during tuning was evaluated using the validation dataset.

The objective functions were defined as balanced accuracy and root mean squared error (RMSE), respectively. Full hyperparameter ranges and final selected values for both BRT models are provided in Appendix S1: Table S2.

After tuning, the final models were refit using the selected parameters. For the WRI model, we assessed feature importance to determine the relative influence of each predictor on survey WRI. In the “gbm3” package, feature importance is computed using the method described by Friedman (2001), which calculates the reduction in prediction error attributable to each variable, averaged over all trees in the model. For each split in a decision tree, the reduction in the loss function (e.g., squared error) is computed for the variable used in that split. These reductions are then aggregated across all trees in the model to give a total reduction in the loss function attributable to each variable. The importance values are then normalized so that they sum to 1, providing a proportional measure of each variable's contribution to predictive performance.

### Neural networks

We implemented a dual‐model NN pipeline to predict survey success and survey WRI. The first NN modeled survey success as a binary outcome and produced the predicted probability that at least one python was captured during a survey. These predicted probabilities were then used as an additional input feature in the second NN that modeled survey WRI. This design allowed the WRI model to account for the conditional dependence of survey WRI on the likelihood of python capture. Two‐part neural architectures that recapitulate a hurdle design are an effective solution for predicting zero‐inflated responses (Kong et al., 2020).

Before fitting, the dataset underwent standard preprocessing for NN models. Rare categorical levels (*n* < 25) were pooled into an “other” category prior to one‐hot encoding. Temporal variables (e.g., survey start and end times, week, month, and Julian day) were converted into sine‐cosine pairs to encode periodicity. All preprocessing steps, including standardization of continuous variables to zero mean and unit variance, were parameterized using the training dataset only and then applied unchanged to the validation and test datasets to avoid information leakage. Both NNs were otherwise fit to the same set of environmental, temporal, and operational predictors described above.

Hyperparameters governing network architecture and regularization were tuned using Bayesian optimization via the “kerastuneR” package (Abdullayev, 2024). Candidate models varied in depth, width, learning rate, dropout, L2 regularization, hidden‐layer activation functions, and whether to include batch normalization layers. The complete search space and final selected values for both NN models are provided in Appendix S1: Table S2. The survey success NN used a binary focal cross‐entropy loss function (α = 0.76, γ = 0) to address class imbalance and included a sigmoid output activation to generate capture probabilities. Validation balanced accuracy served as the objective function for tuning. The survey WRI NN used a Tweedie loss function (*p* = 1.02) with a rectified linear unit (ReLU) output activation to ensure nonnegative continuous predictions, and validation root mean squared error (RMSE) as the tuning objective. All models were trained with the Adam optimizer, batches of size 128, and a maximum of 250 epochs. Training employed early stopping with a patience of 10 epochs based on validation performance. We used model checkpointing to retain and restore the epoch with the best performance for each trial.

For both models, hyperparameters were selected using a fixed validation partition while an independent test partition was reserved exclusively for final evaluation. This approach is consistent with best practices for modern machine learning workflows (Cawley & Talbot, 2010; Goodfellow et al., 2016; Snoek et al., 2012). All data splits were made at the survey‐event level to prevent information leakage between model training and evaluation (Kaufman et al., 2012). Because survey events are spatially and temporally structured, random partitions primarily estimate within‐domain performance for the program represented by the data evaluated here (Roberts et al., 2017).

### Contractor performance

To assess the impact of contractor identity on survey outcomes, we extracted coefficients from the contractor identity random effect term of the survey WRI GAM model. These coefficients quantify the relative contribution of individual contractors to variability in survey WRI, accounting for other random and fixed effects in the model. This analysis allowed us to evaluate whether contractors with higher predicted contributions to survey WRI also exhibited higher realized performance.

We calculated descriptive statistics for each contractor, including the number of captures, total surveys conducted, capture rate (survey success/number surveys), total WRI, and average survey WRI. These statistics provided baseline measures of contractor performance and were used to evaluate the relationship between descriptive metrics and coefficients derived from the survey WRI GAM model. This was accomplished by using a simple linear regression model to determine the relationship between contractor‐specific coefficients and two key performance metrics: average survey WRI and capture rate. For the relationship between contractor coefficients and average survey WRI, a polynomial regression model was fitted to account for potential non‐linear effects, which were not observed in the relationship with capture rate. A simple linear regression model was preferred to more complex frameworks as the covariance structure of the dataset was accounted for during GAM fitting. Using the “stats” package from base R, contractor coefficients were extracted from the GAM model using the *coef* function and linear regression models were fit using the *lm* function (R Core Team, 2024).

Additionally, to compare contractor performance with model‐based forecasts of survey success, we framed each contractor survey as an implicit positive prediction of capture under prevailing environmental and operational conditions. Under this framing, contractor capture rate is directly comparable to model positive predictive value (PPV), which quantifies the proportion of predicted capture events that result in an observed removal. Metrics involving negative predictions (e.g., specificity) cannot be evaluated for contractors because surveys are not conducted under conditions where capture is not expected. Accordingly, PPV provides a common basis for comparing ecological forecasting models to realized human performance.

## RESULTS

### Development of the weighted removal index

**FIGURE 1:**
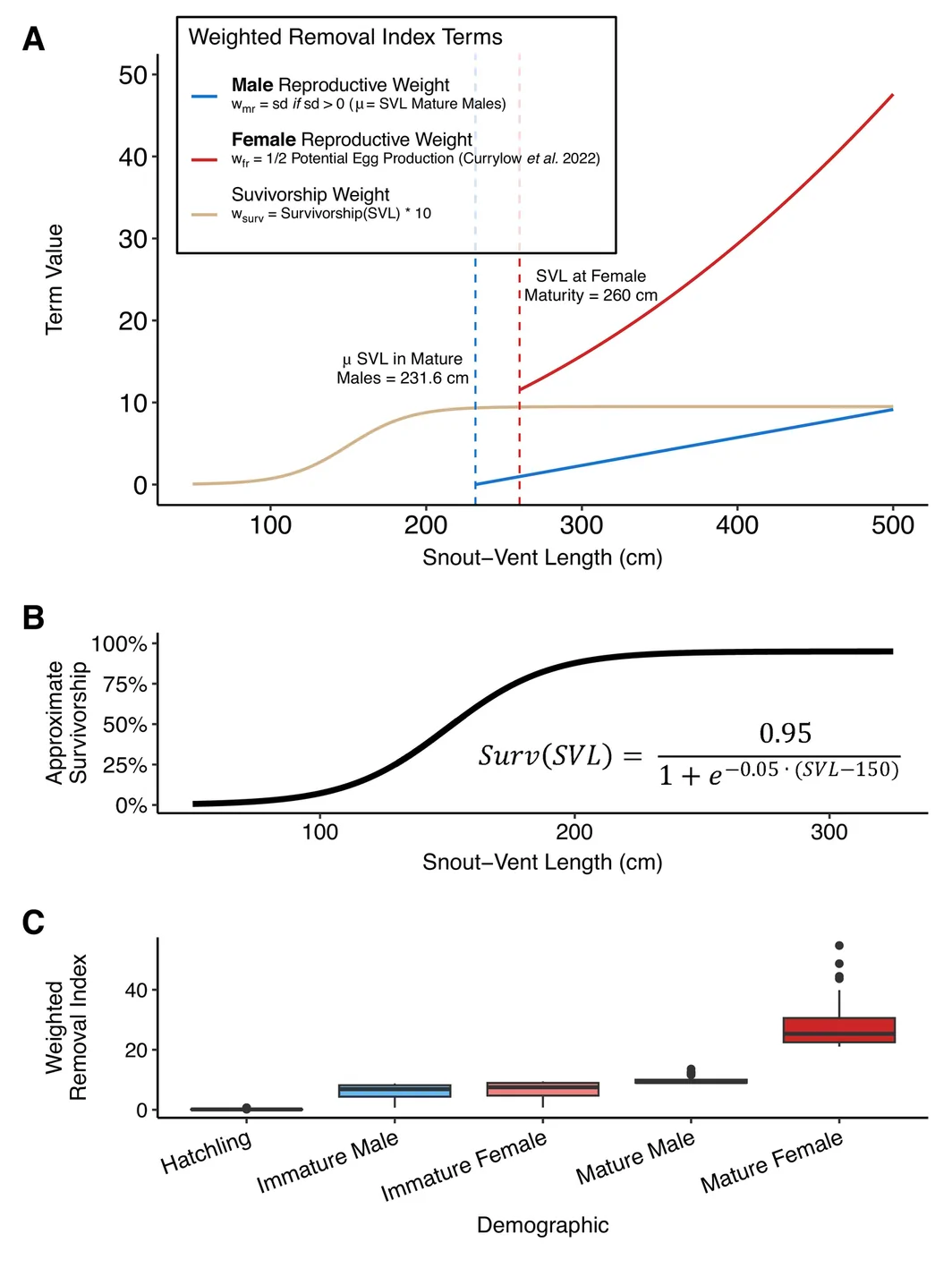
Development and application of the Weighted Removal Index (WRI) for Burmese pythons (*Python bivittatus*). (A) Components of the WRI, including Male Reproductive Weight (blue line), Female Reproductive Weight (red line), and Survivorship Weight (brown line), plotted against SVL. Vertical dashed lines indicate key biological thresholds: mean SVL in mature males (μ = 231.6 cm) and SVL at female maturity (260 cm). (B) Survivorship as a function of snout‐vent length (SVL), showing the modeled relationship between SVL and approximate survivorship based on a sigmoidal function. (C) Boxplot of WRI values across different demographic classes, highlighting the higher WRI in mature females.

The WRI integrates survivorship and reproductive potential to provide a prioritization tool for python removal efforts. The survivorship curve used in this calculation increases with SVL, stabilizing at 95% for pythons exceeding 250 cm in SVL (Figure 1B). The estimates of survivorship produced by this model are consistent with available survivorship data. For example, at SVLs corresponding to approximately 12 months (100 cm) and 24 months (150 cm) of growth (Pittman & Bartoszek, 2021), survivorship estimates of 7.2% and 47.5% are produced. Female reproductive weight, based on potential egg production, increases rapidly with SVL after female maturity (260 cm), as seen in Figure 1A. Male reproductive weight increases linearly with SVL for individuals larger than the population mean of mature males (Figure 1A). The distribution of WRI for all captures in our dataset was visualized according to demographic class (Figure 1C) and SVL (Appendix S1: Figure S1).

To support calculation of the WRI, missing SVL values were imputed using a GAM trained on captures with known SVL. This model demonstrated strong predictive performance, with an adjusted *R* ^2^ of 99.6% and a Mean Absolute Percentage Error (MAPE) of 1.61%, indicating that imputed SVLs closely matched observed values.

**FIGURE 2:**
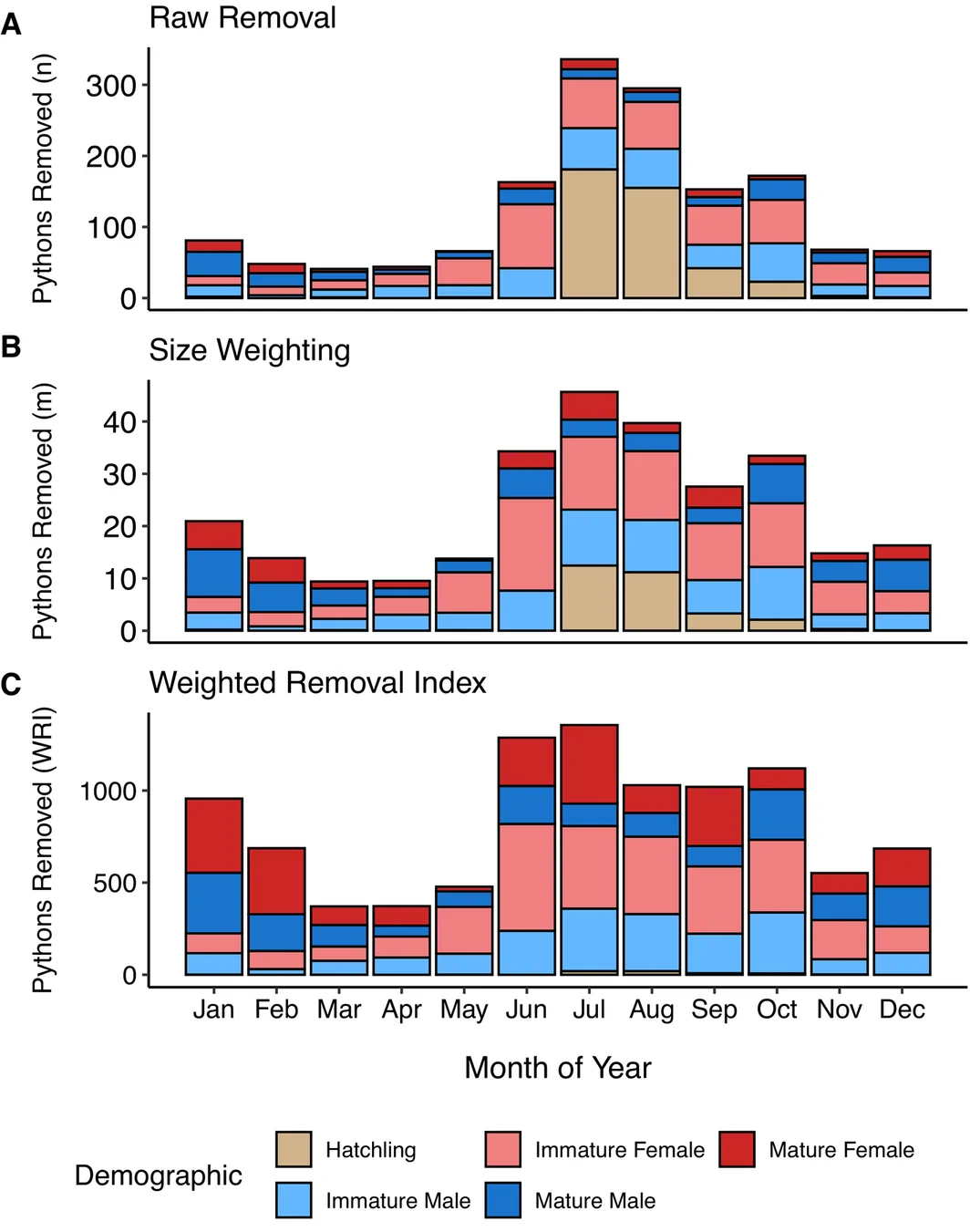
Monthly trends in python removals from the South Florida Water Management District's Python Elimination Program, categorized by demographic class. (A) Raw removal counts by month, showing the total number of snakes removed. (B) Size weighting of removals, calculated by the total length of pythons removed each month (in meters). (C) Weighted Removal Index (WRI) by month, incorporating both survivorship and reproductive potential. Each bar is color‐coded by demographic class: Hatchling (tan; SVL ≤100 cm), Immature Male (light blue; 100< SVL <200 cm), Immature Female (pink; 100< SVL <260 cm), Mature Male (dark blue; SVL ≥200 cm), and Mature Female (red; SVL ≥260 cm). This visualization highlights the differences between raw and demographically weighted removal data, emphasizing the importance of targeting specific life stages for effective population management.

**FIGURE 3:**
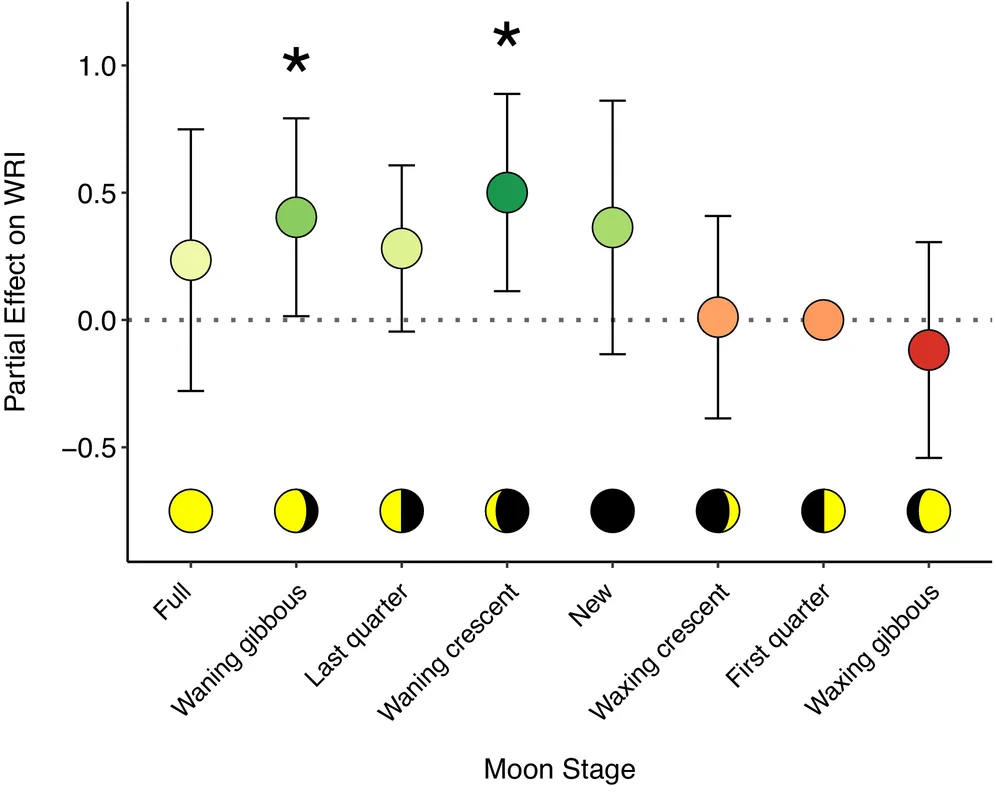
Partial effects of moon stage on the Weighted Removal Index (WRI) for Burmese pythons (*Python bivittatus*) in the South Florida Water Management District's Python Elimination Program. The figure shows the influence of different moon stages on WRI, with larger circles representing greater effects. Moon stages are ordered from full moon to waxing gibbous. The vertical bars represent the 95% CI for each moon stage's effect. Significant positive effects (*p* < 0.05) on WRI were observed during the waning gibbous and waning crescent phases, indicated by asterisks. The partial effects suggest that lunar phases can influence the python removal efforts, with certain phases correlating with higher WRI values. Moon stage illustrations at the bottom of the figure provide a visual representation of each phase.

Python removals showed marked seasonal variation, especially when the demographics of removed individuals were considered. Total removals were highest during summer (June–August), largely due to the high abundance of hatchlings (SVL ≤100 cm) during this period (Figure 2A). Because hatchling captures can bias raw removal totals, we compared three different representations of removal impact: (1) unweighted capture, (2) TL–weighted removals based on the existing PEP incentive structure (Figure 2B), and (3) the WRI (Figure 2C). Incorporating size through total‐length weighting modestly increased the apparent contribution of winter surveys (December–February), but the seasonal pattern remained broadly similar to raw counts. In contrast, the WRI revealed a pronounced seasonal shift: Winter months contributed a disproportionate share of total WRI despite having far fewer captures, reflecting the removal of large, demographically important adults during this period. Summer surveys also remained influential under the WRI framework, but this was due to increased removal of a mixture of mature and immature individuals rather than high hatchling abundance. These comparisons demonstrate that the WRI prioritizes the removal of large, mature individuals more effectively than existing weighting systems.

### Generalized additive models

**FIGURE 4:**
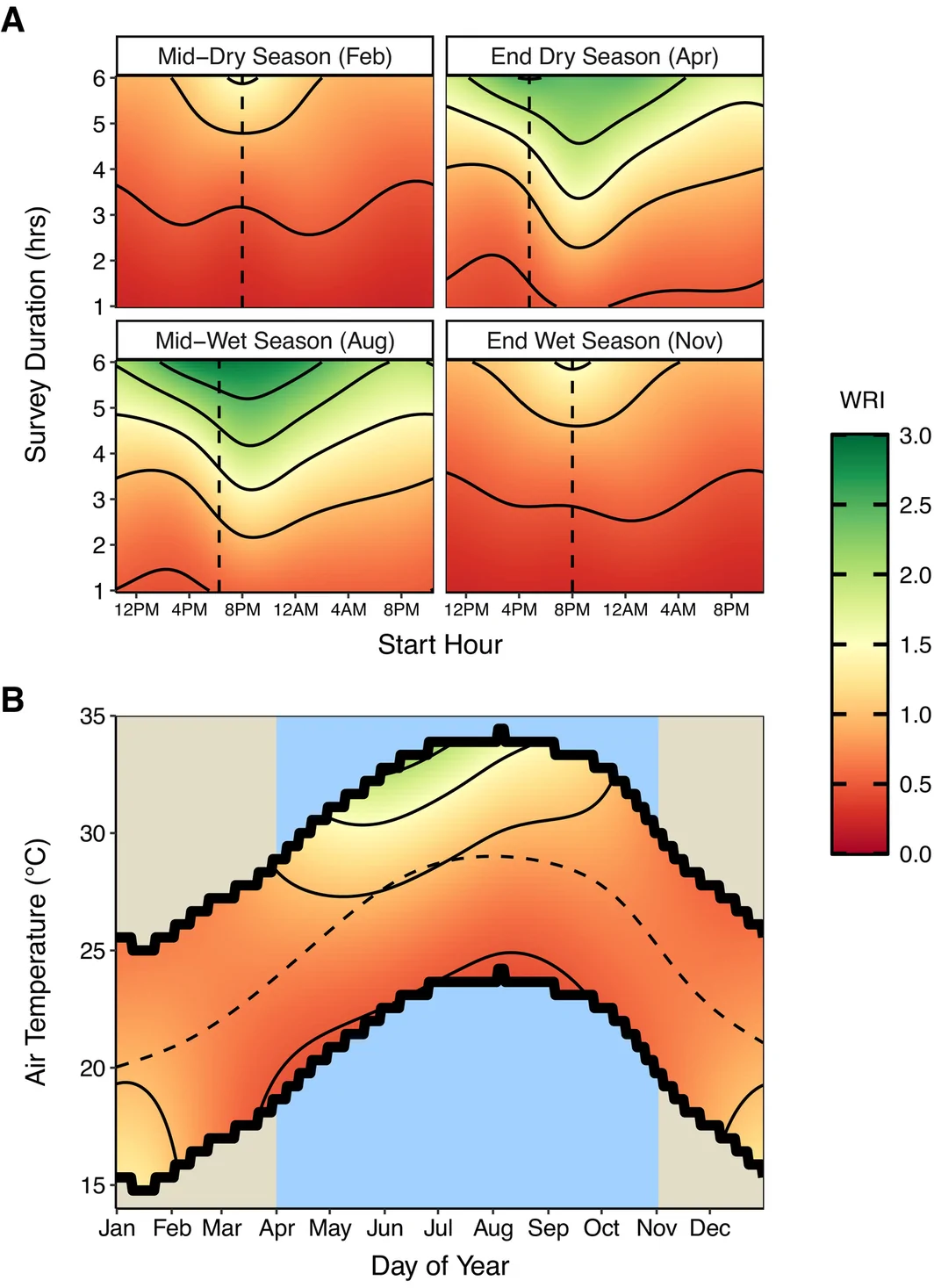
Influence of survey timing and air temperature on Survey Weighted Removal Index (WRI). Only the smooths forming each interaction were allowed to vary; all other terms were held at zero. Thus, fill colors show only the relative effect of the focal interaction. (A) Interaction between survey start hour and survey duration on WRI across four key periods: mid‐dry season (February), end dry season (April), mid‐wet season (August), and end wet season (November). Mean monthly temperature for the corresponding months were utilized in generating model predictions. Warmer colors (red to yellow) indicate lower WRI values, while cooler colors (green) represent higher WRI values. Dashed vertical lines represents the start time with the highest predicted survey WRI. (B) Interaction between air temperature and day of year on WRI. The dashed line represents the seasonal temperature trend, with warmer colors (red to yellow) indicating lower WRI values and cooler colors (green) indicating higher WRI values. Shaded regions indicate tropical seasons: dry (tan) and wet (blue).

We utilized a GAM to assess the influence of various environmental and temporal factors on survey WRI. The final model utilized a Tweedie distribution with a log link and power parameter *p* = 1.1. The model explained 29.8% of deviance in the response. Moon phase was a significant predictor of WRI, with the waning crescent (*p* = 0.0114, γ = 0.50, SE = 0.20, *t* = 2.53) and waning gibbous (*p* = 0.0420, γ = 0.40, SE = 0.20, *t* = 2.03). Thus, surveys conducted under these moon phases will have higher survey WRI (Figure 3). The presence of assistants during surveys also had a significant positive effect on survey WRI (*p* < 0.0001, γ = 0.43, SE = 0.11, *t* = 4.08). Relative humidity (*p* = 0.01569, edf = 0.83, *F* = 0.72) and air temperature (*p* = 0.0059, edf = 0.84, F = 1.27) were positively correlated with survey WRI. Survey interval was a significant predictor of survey WRI, with surveys conducted between 8 PM and 2 AM yielding higher WRI values (*p* < 0.0001, edf = 4.65, *F* = 13.80). However, this effect varied with air temperature (*p* = 0.0324, edf = 4.00, *F* = 0.17). As temperatures increase, surveys that begin earlier in the evening and last longer tend to yield higher WRI values (Figure 4A). Julian day also had a significant effect on survey WRI (*p* = 0.0120, edf = 3.04, *F* = 0.69). The partial effect of Julian day was lowest at the peak of the summer season, likely due to the abundance of hatchlings on the landscape. Additionally, an interaction between Julian day and air temperature (*p* < 0.0001, edf = 2.54, *F* = 2.22) further highlights the complexity of seasonal effects. As shown in Figure 4B, survey WRI values are increased on warm days in early summer, coinciding with hatchling emergence. Alternatively, survey WRI is increased on cool days in the dry season, likely reflecting increased capture of basking adult pythons. These findings emphasize that while survey timing plays a key role in removal success, its effect is mediated by temperature rather than being strictly seasonal.

The model identified several significant random effects. Notably, survey sites Stormwater Treatment Area 3/4 (*p* = 0.0052, edf = 0.87, *F* = 148.55) and Rotenberger Wildlife Management Area (*p* = 0.0007, edf = 0.91, *F* = 29.14) had significant effects on survey WRI, suggesting that demographics of captured pythons are not evenly distributed across sites. Contractor identity was also a significant random effect (*p* < 0.0001, edf = 0.39, *F* = 5.34), suggesting differences in skill across contractors. Finally, survey type was a significant source of variation in survey WRI (*p* < 0.0001, edf = 2.81, *F* = 7.36). Survey transport method was included as a random intercept term, allowing estimation of its association with survey WRI after accounting for the other modeled predictors. Motorboats (β = 0.75), bikes (β = 0.27), and airboats (β = 0.16) had positive coefficients, indicating higher‐than‐average survey WRI on surveys conducted using these methods. These coefficients should be interpreted as operational associations rather than causal estimates of method effectiveness.

### Boosted regression trees

**FIGURE 5:**
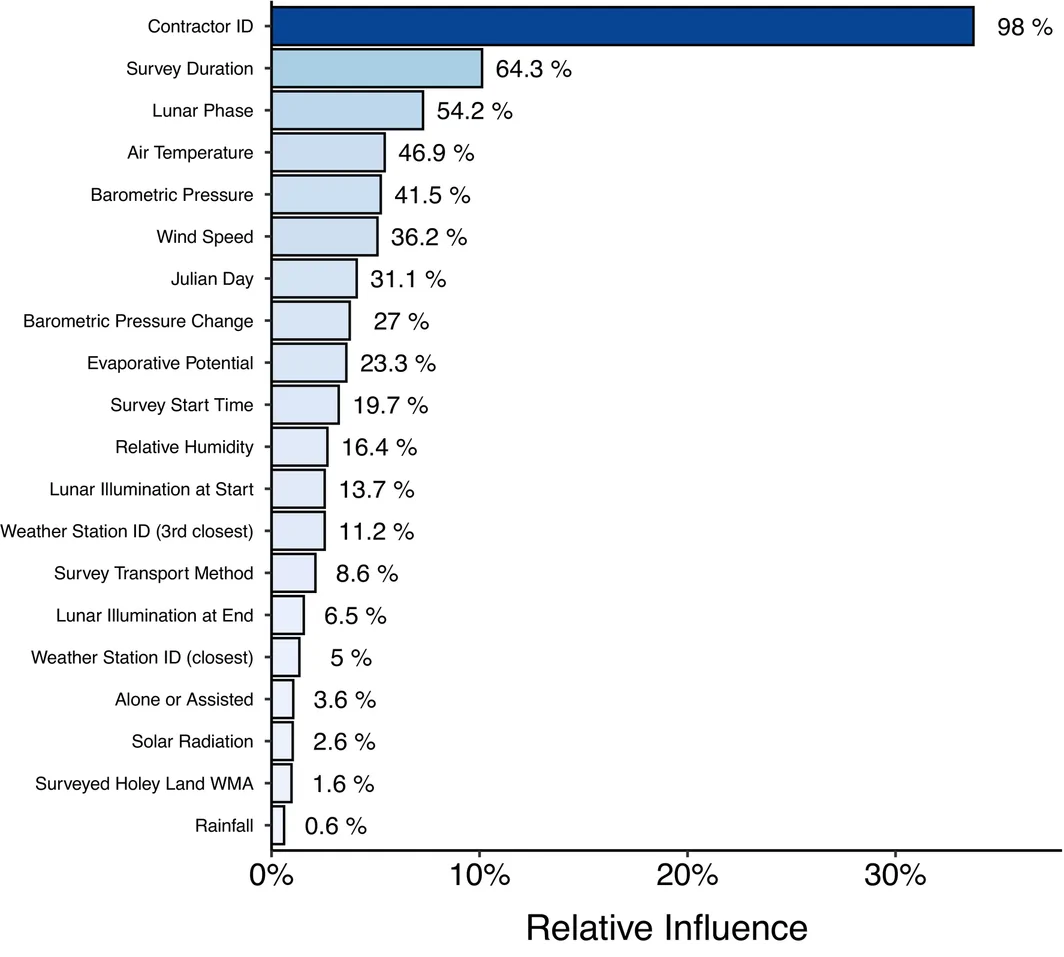
Relative influence of predictor variables on survey Weighted Removal Index (WRI) in the South Florida Water Management District's Python Elimination Program, as determined by the Boosted Regression Trees (BRT) model. Contractor identity was the most influential predictor, contributing 33.7% to model performance, followed by Survey Duration (10.1%) and Lunar Phase (7.3%). Other important factors included air temperature (5.4%), barometric pressure (5.3%), wind speed (5.1%), and Julian day (4.1%). These findings highlight the importance of both contractor‐specific factors and environmental conditions in determining survey success and WRI. Percentages at the end of each bar represent the cumulative sum of relative influence.

We applied a BRT model to evaluate the influence of various environmental and operational factors on survey WRI. Contractor identity was identified as the most influential predictor, contributing 33.7% to the model's predictive performance, potentially reflecting differences in skill or methodology. Operational variables like survey duration (10.1%), survey start time (3.2%), Julian day (4.1%), and survey transport method (2.1%) were also important, suggesting that timing and logistics affect survey outcomes. Environmental factors, including air temperature (5.4%), barometric pressure (5.3%), wind speed (5.1%), barometric pressure change (3.8%), evaporative potential (3.6%), and relative humidity (2.7%), influenced predictions of survey outcomes, while rainfall (0.6%) had minimal influence. Finally, lunar‐related variables, such as lunar phase (7.3%) and lunar illumination at start (2.6%), also played an important role in predicting survey WRI. Figure 5 presents the relative influence of the top 20 predictors.

### Neural networks

The NN analysis was conducted using a dual‐model pipeline as described in the methods, where predicted survey success was used as an input feature for the survey WRI model. Details of the tuning process, including the search space and selected architectures for each model, are reported in Appendix S1: Table S2. NN performance across both outcome classes is evaluated alongside the other model classes.

### Model performance

Model performance was evaluated on the independent test dataset for each outcome (survey success and survey WRI) and modeling framework. Because survey outcomes were strongly imbalanced (76% no capture), we emphasize balanced accuracy, the mean of sensitivity and specificity, as an overall metric of classification model performance. Balanced accuracy deemphasizes prediction of the majority class, which reduces bias associated with traditional measures of model performance (e.g., area under the ROC curve [AUC]) for unbalanced ecological datasets (Shabani et al., 2018).

Balanced accuracy was highest for the NN (70.3%), followed by BRT (66.7%) and GAM (63.9%). This indicates that NN achieved the strongest balance between correctly identifying captures and non‐captures. However, examination of sensitivity and specificity of each model reveals differences in prediction bias toward each outcome class. The NN had the highest sensitivity (65.8%), indicating it identified the largest proportion of true capture events, whereas the BRT and GAM had sensitivities of 56.7% and 36.7%, respectively. In contrast, the GAM had the highest specificity (91.1%), exceeding the BRT (76.7%) and NN (74.7%), indicating that the GAM most effectively reduced false positives. Together, this suggests the NN was more aggressive in flagging potential captures (capturing more true positives), while the GAM was more conservative (more reliably avoiding non‐capture scenarios).

For survey WRI, predictive error was similar across approaches. The NN yielded the lowest root mean squared error (RMSE = 5.50), compared with BRT (5.54) and GAM (5.59). Additionally, the NN had the highest Kendall correlation (0.37), followed by BRT (0.36) and GAM (0.29), indicating that the NN most consistently preserved the rank ordering of surveys by predicted WRI, which is central to prioritization.

To facilitate comparison of model performance, performance metrics for each model and outcome are summarized in Table 1.

**TABLE 1:** Predictive performance of models for survey success (binary classification) and survey Weighted Removal Index (WRI; regression), evaluated on a held‐out test set.

Model	Sensitivity	Specificity	Balanced accuracy	PPV	NPV	AUC	MAE	RMSE	Kendall's τ
GAM	0.37	**0.91**	0.64	**0.56**	0.82	**0.77**			
BRT	0.57	0.77	0.67	0.43	0.85	0.67			
NN	**0.66**	0.75	**0.70**	0.44	**0.88**	0.70			
GAM							3.15	5.59	0.29
BRT							**2.79**	5.54	0.36
NN							2.99	**5.50**	**0.37**

*Note:* Bold values indicate the strongest performance value (maxima or minima) for that metric.

Abbreviations: AUC, area under the receiver operating characteristic curve; BRT, boosted regression tree; GAM, generalized additive model; MAE, mean absolute error; NN, neural network; NPV, negative predictive value; PPV, positive predictive value; RMSE, root mean squared error; WRI, Weighted Removal Index.

### Contractor performance

**FIGURE 6:**
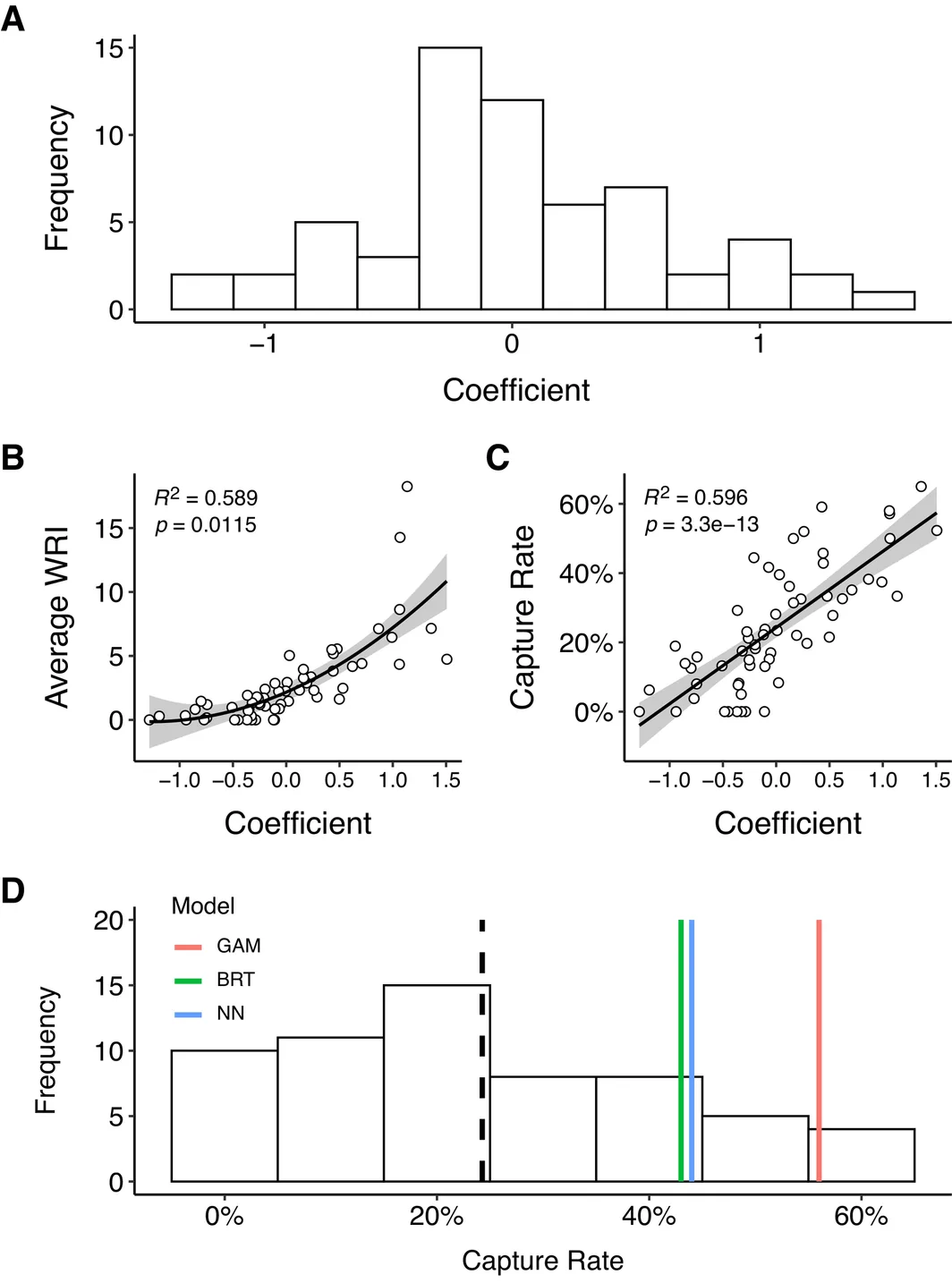
Analysis of contractor performance from South Florida Water Management District's Python Elimination Program. (A) Histogram of contractor coefficients derived from the Generalized Additive Model (GAM). (B) Relationship between contractor coefficients and average survey Weighted Removal Index (WRI). The linear regression indicates a significant positive correlation (*R* ^2^ = 0.589, *p* = 0.0115), suggesting that contractors with higher model coefficients yield higher WRI values on average. (C) Relationship between contractor coefficients and capture rate. The linear regression shows a significant positive correlation (*R* ^2^ = 0.596, *p* = 3.3e‐13), indicating that contractors with higher coefficients achieve greater capture rates. (D) Distribution of contractor capture rates, with vertical solid lines indicating the positive predictive value (PPV; analogous to capture rate) of survey success models, and the dashed vertical line indicating the mean capture rate across all contractors.

The distribution of contractor coefficients derived from the GAM model of survey WRI ranged from −1.28 to 1.51 (Figure 6A), revealing substantial variability in contractor performance. The median contractor coefficient was −0.11, with the first quartile at −0.35 and the third quartile at 0.43. Thus, while many contractors contribute modestly to survey outcomes, there are significant outliers, both positively and negatively.

There was a significant positive correlation between contractor coefficients and average survey WRI (*R* ^2^ = 0.589, *p* = 0.0115; Figure 6B), indicating that contractors with higher model coefficients achieve higher average survey WRI in practice. Additionally, the correlation between contractor coefficients and capture rates (*R* ^2^ = 0.596, *p* = 3.3e‐13; Figure 6C) further supports this metric of contractor performance, as contractors with higher coefficients also demonstrate higher capture rates.

Across contractors, capture rates were highly variable (median = 0.22, interquartile range = 0.13–0.36). Model PPVs fell in the upper tail of the contractor capture‐rate distribution. Specifically, the NN and BRT PPV values (0.44 and 0.43, respectively) exceeded 84% of observed contractor capture rates, while the GAM PPV (0.56) exceeded 93%. This indicates that when models predicted capture, their reliability was comparable to that of the highest performing contractors under typical conditions (Figure 6D).

## DISCUSSION

Burmese pythons are highly cryptic. Consequently, removal programs for this species benefit from decision‐support tools that prioritize effort toward high‐impact removals, rather than treating all captures as equivalent. In this study, we introduced the WRI, a demographic weighting framework that prioritizes removals by survivorship and reproductive potential. We show that applying demographic weighting can change prioritization relative to unweighted removal or exclusively size‐based weighting. While seasonal patterns in python activity and encounters are well documented (Bartoszek et al., 2021; Guzy et al., 2023), our results demonstrate that removals during these periods, particularly during the breeding season, generate disproportionate demographic impacts. Additionally, we identify operational and environmental conditions associated with higher survey WRI. Survey WRI was elevated under waning crescent and waning gibbous moons, when surveys were assisted, and under warmer and more humid conditions. Survey WRI was elevated on warm days early in the wet season, prior to hatchling emergence, but decreased following emergence. Survey WRI also increased on cool dry‐season days, consistent with adult basking behavior. Finally, we show that ecological forecasting models can predict survey success with greater reliability than baseline contractor performance, supporting the use of near‐term forecasting as a decision‐support tool in cryptic invader management where detection probabilities are low (Pichler & Hartig, 2023).

Demographic weighting of pythons builds on concepts from population ecology, which emphasize that specific life stages contribute disproportionately to population growth, making them effective targets for management actions (Crouse et al., 1987). In invasive species control, targeting demographic classes can improve management efficiency relative to strategies that treat all individuals equivalently (Buhle et al., 2005; Loppnow & Venturelli, 2014). For highly cryptic invaders, such as Burmese pythons, demographic weighting allows managers to evaluate removal targets even when absolute population size is uncertain. The WRI operationalizes this idea by weighting each removal by expected demographic impact, providing a relevant metric for comparing surveys, contractors, and time periods. Thus, demographic removal can be integrated with functional eradication, where the objective is to reduce invader populations to levels associated with acceptable ecological effects rather than pursuing complete eradication (Green & Grosholz, 2021). For pythons, this could support management targets (e.g., annual WRI targets) which could be evaluated against recovery criteria (e.g., mammal survey data) to facilitate functional eradication. Additionally, the application of near‐term forecasting can improve detection, prioritization, and overall management efficiency.

NNs have shown great promise in predicting species presence (Deneu et al., 2021). This is particularly important for invasive species management, where increasing detection efficiency can facilitate additional removal and thereby reduce associated ecological impacts (Lieurance et al., 2023). Our study demonstrated that NN models can enhance the capacity to forecast both the likelihood of capturing pythons and the potential impact of those removals. By improving detection accuracy and guiding removal toward individuals with high reproductive output, NNs can optimize resource allocation. For example, managers can use NN predictions to inform contractors of conditions and localities that are predicted to maximize expected WRI per survey hour. Future implementations of this technology could generate these predictions in real time and distribute them via a smartphone application. This would allow contractors to make data‐driven decisions in the field and align desired management outcomes more closely with allocated effort.

Such an approach aligns with recent trends in ecological research, where machine learning techniques are increasingly being used to address complex, nonlinear relationships in species distribution and detection models (Brodrick et al., 2019; Deneu et al., 2021). Additionally, monitoring shifts in prey populations or ecosystem function after targeted removals would provide evidence of broader ecological benefits associated with this approach; thereby enabling managers to better assess the success of python management programs.

By incorporating demographic characteristics, the WRI offers a potentially more effective approach to prioritizing python removals beyond raw removal counts or TL. For example, the WRI could be considered when designing the incentive structure of contractor programs. Because the WRI increases nonlinearly with size and is higher for reproductive individuals, such a structure might reward removal of one mature female more highly than other demographics. This may incentivize contractors to allocate effort toward conditions associated with higher predicted survey WRI, rather than maximizing encounter counts alone. As shown in Figure 2, demographic weighting can shift the relative importance of removals across months, emphasizing periods when mature individuals contribute a larger fraction of total WRI. Notably, lunar phase emerged as a significant predictor of survey WRI, which is consistent with evidence that moonlight can influence nocturnal activity in snakes (Clarke et al., 1996). However, lunar phase was not recovered as a significant predictor of unweighted removals in PEP data (McCaffrey et al., 2025), suggesting that lunar conditions may have demographic‐specific effects on removal. Furthermore, this demographic prioritization framework could be incorporated with existing geographic prioritization to allocate effort along the invasion curve (Harvey & Mazzotti, 2015).

Our findings also highlight the significant variability in contractor performance, with some contractors consistently achieving higher survey success and WRI values than others. This is consistent with previous work: Analyses of the SFWMD program identified contractor identity as a major determinant of survey outcomes (McCaffrey et al., 2025), and volunteer efforts in Everglades National Park showed strong interindividual heterogeneity (Falk et al., 2016). This variability suggests that contractor identity plays a crucial role in the outcomes of python removal efforts. Importantly, the connection between predicted and actual performance highlights the utility of the GAM‐derived coefficients as a predictive tool for contractor effectiveness. To address this, management agencies could consider implementing standardized training programs based on the techniques of high‐performing contractors. These findings underscore the importance of contractor skill and suggest that contractor removal performance should be considered when designing removal efforts to maximize the effectiveness of survey outcomes. Importantly, the high influence of contractor identity, observed across multiple models, suggests a need for standardized training or protocols to reduce variability in survey outcomes.

Currently, the WRI relies on several key assumptions that should be acknowledged, particularly the survivorship of Burmese pythons. The survivorship curve we used is an approximation derived from a combination of limited empirical data and expert opinion, as survivorship data for Burmese pythons in Florida is limited in scope. Juvenile survivorship estimates are informed by field data (Pittman & Bartoszek, 2021), whereas adult survival has been parameterized in previous models using expert opinion (Willson et al., 2011). While our model estimates are consistent with published survivorship values, further empirical research is needed to refine these assumptions and improve the accuracy of the WRI. Additionally, the reproductive weights assigned to pythons are based on known life history attributes of snakes, but Burmese pythons display plastic reproductive characteristics (Currylow et al., 2022a). Studies that provide additional data regarding the life history of Burmese pythons in South Florida could improve future versions of the WRI. Additionally, male reproductive weights are calculated based on population‐level statistics rather than empirical relationships between size and reproductive output in Burmese pythons. This introduces uncertainty, as it assumes a consistent relationship between male size and reproductive success across individuals and environmental contexts. While this approach is informed by analogous studies in other snake species, the lack of species‐specific data may under‐ or overestimate the contribution of large males to population dynamics. Field studies aimed at quantifying reproductive success among male Burmese pythons of varying sizes would help validate this assumption and refine demographic weighting within the WRI framework. Furthermore, this demographic weighting scheme assumes that removal mortality is additive at the population level and does not result in compensatory mortality or overcompensation. If such density‐dependent responses occur, the benefits of targeted removal could be reduced (Guzy et al., 2023). Finally, because we did not implement spatial or temporal blocking in model evaluation, reported test performance should be interpreted as within‐program generalizability, reflecting predictive performance for surveys drawn from the same monitoring domain rather than extrapolation to novel properties or time periods (Roberts et al., 2017). To ensure these tools effectively guide python removal efforts, we recommend targeted validation of the WRI and machine learning models through empirical field testing. While our results suggest that these tools can improve management outcomes, ongoing monitoring and evaluation will be necessary to assess their real‐world impact over time. Longitudinal studies that determine population responses to WRI‐guided removal will be essential for evaluating its efficacy, particularly for functional eradication objectives.

Building on the development and application of the WRI and machine learning models, future research should focus on refining these tools, expanding their applicability, and validating their effectiveness in management scenarios. Enhancing the accuracy of the WRI will require more detailed demographic data, and including hydrological variables, measures of human development, and python movement data in NN models of the WRI may improve their predictive capacity. Integrating the WRI into management programs with systematic tracking of outcomes would allow for comparisons between demographic‐based prioritization and traditional removal strategies. Such integration would also help clarify the added value of these tools in meeting broader management goals. Finally, exploring the human dimensions of invasive species management, such as contractor training and willingness to adopt new techniques, is crucial. Developing best practices for training and standardizing protocols based on high‐performing individuals could help reduce variability and maximize the efficiency of removal efforts.

Through the development and application of the WRI and advanced machine learning models, we present a novel framework for managing a cryptic invasive species. This demographic‐removal strategy offers a targeted approach to invasive species management which attempts to prioritize removals according to impacts on population vital rates. Our findings also demonstrate that NNs, with their ability to capture complex, nonlinear relationships, can enhance predictive capacity in invasive species management. The implications of our findings extend beyond the context of Burmese python management. The WRI framework and the use of machine learning models offer valuable insights for managing other invasive species, particularly those with low detectability and demographic‐specific survivorship. By prioritizing individuals that contribute disproportionately to population growth with predictive modeling, management agencies can maximize the effectiveness of control efforts. Comparative studies that apply the WRI framework to multiple species could help identify commonalities and differences in invasion dynamics, guiding the development of more generalizable management tools. By refining the WRI, expanding its application, and integrating long‐term monitoring and human factors, future research can contribute to more effective invasive species management strategies.

## AUTHOR CONTRIBUTIONS

Frank J. Mazzotti and Melissa A. Miller conceptualized the project and obtained funding. Michael Kirkland, Edward F. Metzger III, Amy Peters, and LeRoy Rodgers provided data. Alexander S. Romer, Edward F. Metzger III, and Amy Peters conducted data management and curation. Alexander S. Romer performed statistical modeling. Melissa A. Miller, Sergio A. Balaguera‐Reina, Alexander S. Romer, and Frank J. Mazzotti drafted the initial manuscript. All authors contributed to revisions.

## CONFLICT OF INTEREST STATEMENT

The authors declare no conflicts of interest.

## Supporting information


Appendix S1.

## Data Availability

Data and code (Romer, 2026; Romer et al., 2026) are available in Zenodo at https://doi.org/10.5281/zenodo.21416777. The following data were also used in this research: Currylow et al. (2022b), available in a U.S. Geological Survey data release https://doi.org/10.5066/P9CZI2KO; Palecki et al. (2021) (subset: 1991–2020 daily air‐temperature normals for Miami International Airport, Florida), available in the NOAA National Centers for Environmental Information at https://www.ncei.noaa.gov/access/metadata/landing-page/bin/iso?id=gov.noaa.ncdc:C01621.
