# Supplementary material for: Prioritizing removals of a highly cryptic invasive species using machine learning and demographic weighting
Source: Ecol Appl. 2026 Sep 21;36(6):e70317. doi: 10.1002/eap.70317 (PMC13593391; doi:10.1002/eap.70317)
Supplement: Supplementary file 1 [file EAP-36-e70317-s001.pdf]

Appendix S1

Prioritizing removals of a highly cryptic invasive species using machine learning and demographic weighting

Alexander S. Romer, Melissa A. Miller, Sergio A. Balaguera-Reina, Michelle C. Bassis, Brandon Welty, Michael Kirkland, Amy Peters, Edward F. Metzger III, Jenna Cole, LeRoy Rodgers, and Frank J. Mazzotti

Ecological Applications

Any use of trade, firm, or product names within this manuscript is solely for descriptive purposes and does not imply endorsement by the U.S. Government.

Section S1: Tables

Table S1 (next page). Definitions and descriptions of covariates used in machine learning and statistical models to predict python encounter probability and removal quality. Variables encompass operational, temporal, lunar, and environmental predictors used to model python survey success and demographic impact (Weighted Removal Index, WRI). Abbreviations correspond to variable names in the modeling dataset, with units provided where applicable.

Appendix S1

Abbreviation	Variable	Description
airt.c	Air Temperature	Daily mean air temperature (°C)
alone_or_assist	Alone or Assisted	Two level factor identifying if a contractor was alone or assisted when conducting a survey
baro.change	Barometric Trend	Categorical trend in barometric pressure (positive, negative, same)
baro.diff	Change in Barometric Pressure	Change in daily mean barometric pressure from previous day (mm Mercury)
baro.mm	Barometric Pressure	Daily mean barometric pressure (mm Mercury)
contractor	Contractor Identifier	Unique identifier assigned to each PEP contractor
end_minutes	End Time	End time of survey in minutes from midnight (0–1439)
etp.mm	Evaporative Potential	Daily cumulative evaporative potential (mm)
humi.perc	Relative Humidity	Daily mean relative humidity (%)
illum_end	Lunar Illumination at End	Proportion of the moon illuminated at survey end
illum_start	Lunar Illumination at Start	Proportion of the moon illuminated at survey start
julian	Julian Day	Julian day of survey (1–366)
month.n	Month	Numeric month of survey (1–12)
moon_end4	Lunar Phase End (4 levels)	Lunar phase at survey end as one of: full, new, waxing, waning
moon_end8	Lunar Phase End (8 levels)	Lunar phase at survey end as one of: first quarter, full, last quarter, new, waning crescent, waxing gibbous, waxing crescent, waning gibbous
moon_phases	Moon Phase (Trend)	Lunar phase at survey start as one of: crescent, full, gibbous, new, quarter
moon_start4	Lunar Phase Start (4 levels)	Lunar phase at survey start as one of: full, new, waxing, waning
moon_start8	Lunar Phase Start (8 levels)	Lunar phase at survey start as one of: first quarter, full, last quarter, new, waning crescent, waxing gibbous, waxing crescent, waning gibbous
PATRIC_property_equiv	PATRIC Property Equivalent	Unique identifier for each PATRIC Property Equivalent
radt.kwm2	Solar Radiation	Daily mean total solar radiation (kw/m²)
rain.mm	Total Rainfall	Daily cumulative rainfall (mm)
season2	Tropical Seasons	Wet (May – October) and Dry (November – April)
season4	Temperate Seasons	Spring (March – May), Summer (June – August), Fall (September – November), and Winter (December – February)
start_minutes	Start Time	Start time of survey in minutes from midnight (0–1439)
station1	Weather Station 1	Primary weather station identifier
station2	Weather Station 2	Secondary weather station identifier
station3	Weather Station 3	Tertiary weather station identifier
survey_hr	Survey Duration	Length of survey (hours)

Appendix S1

survey_type	Survey Methodology	Transportation method used to conduct survey (e.g., driving, walking, airboat)
week	Week	Numeric Week of the calendar year (1–53)
wnds.mph	Wind Speed	Daily mean wind speed (mph)
year	Year	Calendar year of the survey (e.g., 2020, 2021, etc.)

Table S2. Hyperparameter search space and selected tuning values for boosted regression tree (BRT) and neural network (NN) models used to predict survey success (binary capture outcome) and survey Weighted Removal Index (WRI). For each model and outcome, the table lists the grid or range of candidate values evaluated, the final selected value for each outcome, and brief notes describing the role of each hyperparameter. Selected values reflect those minimizing validation error or maximizing predictive performance during model tuning.

Model	Outcome	Hyperparameter	Parameter Values	Selected Value		Description
				Survey Success	Survey WRI	
BRT	Both	n.trees	500, 1000, 5000	500	1000	Number of boosting iterations (grid search range)
		interaction.depth	3, 5, 7	5	7	Tree interaction depth (maximum splits per tree)
		shrinkage	0.005, 0.01, 0.05, 0.1	0.1	0.01	Learning rate values in grid search
		n.minobsinnode	5, 10, 15	10	5	Minimum observations per terminal node
		training_weights	1, 2, 3	3	1	Multiplicative weight applied to capture events
NN	Both	depth	1–5	5	1	Number of hidden layers
		units_per_layer	16, 32, 64, 128, 256, 512	64, 128, 512, 16, 32	256	Candidate neurons per hidden layer
		dropout	0–0.3 (step 0.05)	0.1, 0.1, 0.1, 0.1, 0.1	0.15	Dropout rate applied after each hidden layer
		learning_rate	$1e^{-6}$–$1e^{-2}$ (log scale)	$1.0e^{-4}$	$1.0e^{-3}$	Adam learning rate
		L2_regularization	$1e^{-6}$–$1e^{-2}$ (log scale)	$3.5e^{-4}$	$3.6e^{-4}$	kernel L2 regularization strength
		activation_hidden	relu, elu	elu	relu	Candidate activation functions in hidden layers
		batch_normalization	TRUE, FALSE	TRUE	FALSE	Incorporate batch normalization after dense layers
	WRI	Tweedie_p	1.01–1.99		1.02	Power parameter (*p*) for Tweedie loss

Section S2: Equations

Equation S1 (next page). Formula specification provided to `*mgcv*` for WRI model. Note that variables indicated with blue text are dummy variables created from the `*PATRIC_property_equiv*` field. Sites with ≥ 25 surveys were converted to binary factors for each survey (1 = surveyed at site; 0 = did not survey site). A composite variable (`*OtherSites*`) indicates whether a survey passed through any site below this threshold.

```
wri ~ moon_start8 + season2 + alone_or_assist +
 s(airt.c, bs = "tp", k = 10) +
 s(julian, bs = "cc", k = 18) +
 ti(julian, airt.c, bs = c("cc", "ts")) +
 ti(start_minutes, survey_hr, bs = c("cc", "ts")) +
 ti(start_minutes, survey_hr, airt.c, bs = c("cc", "ts", "ts")) +
 ti(start_minutes, survey_hr, julian, bs = c("cc", "ts", "cc")) +
 s(baro.diff, by = season2, bs = "ts") +
 s(wnds.mph, bs = "ts") +
 s(radt.kwm2, bs = "ts") +
 s(etp.mm, bs = "ts") +
 s(humi.perc, bs = "ts") +
 s(baro.mm, bs = "ts") +
 s(rain.mm, bs = "ts", k = 10) +
 te(illum_start, illum_end, bs = c("ts", "ts")) +
 s(station1, station2, station3, bs = "re") +
 s(contractor, bs = "re") +
 s(year, bs = "re") +
 s(survey_type, bs = "re") +
 s(SG, bs = "re") +
 s(FP_RG, bs = "re") +
 s(EVER, bs = "re") +
 s(RG, bs = "re") +
 s(FP, bs = "re") +
 s(BBCW, bs = "re") +
 s(PENN, bs = "re") +
 s(OtherSites, bs = "re") +
 s(SMA_8.5, bs = "re") +
 s(L31N, bs = "re") +
 s(ROTE, bs = "re") +
 s(STA3_4, bs = "re") +
 s(HOLY, bs = "re") +
 s(FST, bs = "re") +
 s(BICY, bs = "re") +
 s(FSPSP, bs = "re") +
 s(L31E, bs = "re") +
 s(BISC, bs = "re")
```

Section S3: Figures

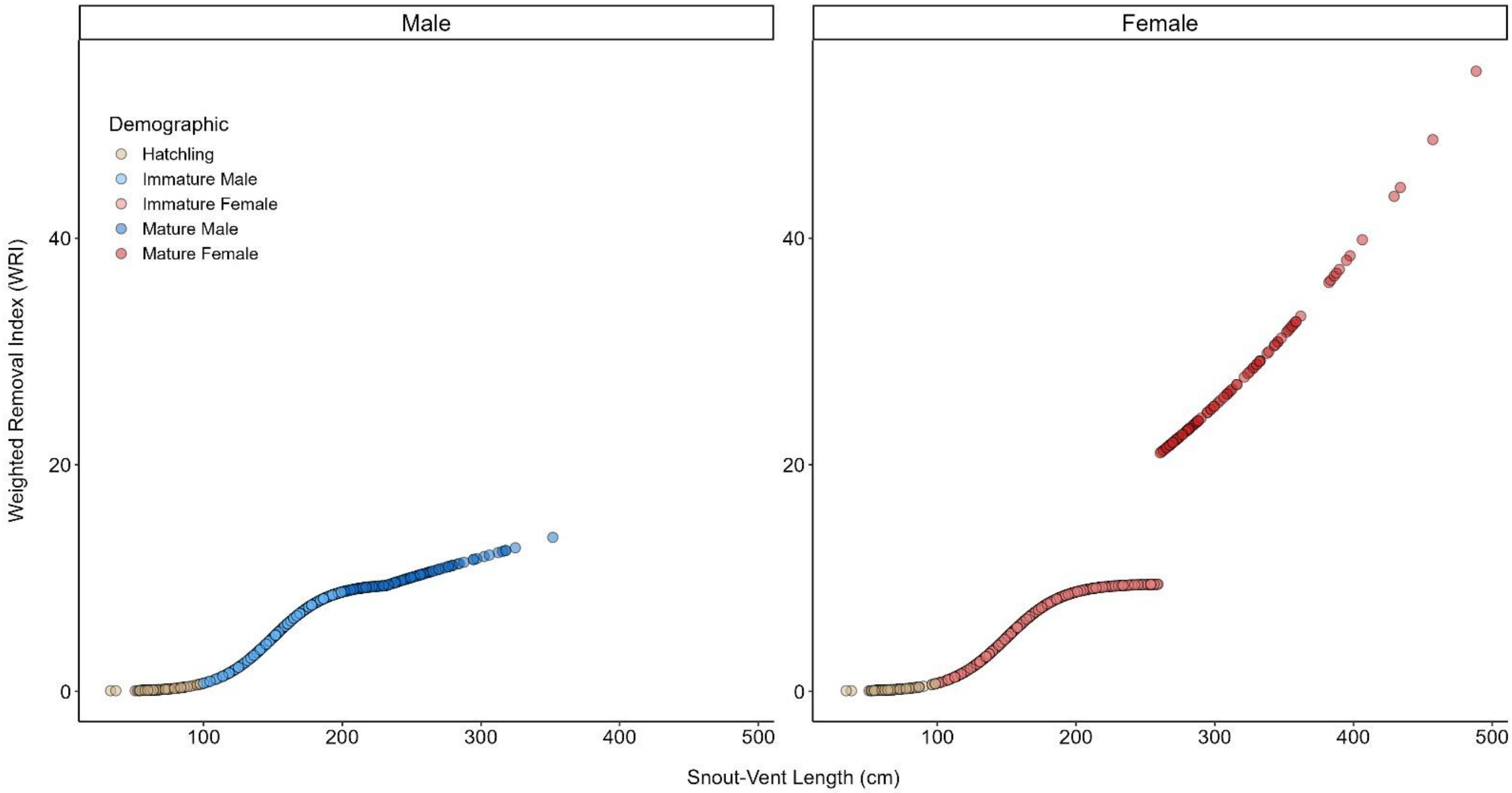

Figure S1. Scatter plots showing WRI as a function of SVL for males (left) and females (right) in our dataset, with individuals categorized by demographic class (e.g., hatchling, immature, mature). These visualizations illustrate how the WRI prioritizes the removal of individuals that contribute disproportionately to population growth, particularly larger, mature females.
